# Spin-polarized DFT study of Pr_2_EuMO_6_ (M = Co, Fe) double perovskites for spintronic and energy applications

**DOI:** 10.1039/d6ra01748g

**Published:** 2026-05-12

**Authors:** Ahmad Ali, Gulzar Khan, Tania Gul, Sikander Azam, Fareha, Osama Oqilat, Hijaz Ahmad

**Affiliations:** a Department of Physics, Government Degree College Lahor Swabi Pakistan aaphy12@gmail.com; b Department of Physics, Abdul Wali Khan University Mardan 23200 Pakistan; c University of West Bohemia, New Technologies Research Centre 8 Univerzitní Pilsen 306 14 Czech Republic azam@ntc.zcu.cz; d Faculty of Engineering and Applied Sciences, Department of Physics, Riphah International University Islamabad Pakistan; e Department of Basic Sciences, Faculty of Arts and Science, Hourani Center for Applied Scientific Research, Al-Ahliyya Amman University Amman Jordan; f Irfan Suat Gunsel Operational Research Institute, Near East University, Nicosia/TRNC 99138 Mersin 10 Turkey hijaz.ahmad@neu.edu.tr; g Department of Mathematics, College of Science, Korea University 145 Anam-ro, Seongbuk-gu Seoul 02841 South Korea; h Engineered Biomaterials Research Center, Khazar University Baku Azerbaijan

## Abstract

Spin-polarized DFT-based computations have been performed using mBJ + U approximations to explore the structural, optoelectronic, magnetic, and thermoelectric characteristics of Pr_2_EuMO_6_ (M = Co, Fe) materials in the cubic (*Fm*3̄*m*) space group. The study has made the materials promising in spintronic applications due to their half-metallic nature. Magnetic analysis shows that the materials are ferromagnetic. The overall magnetic moments of Pr_2_EuCoO_6_ and Pr_2_EuFeO_6_ are 11 (*µ*_B_) and 14 (*µ*_B_), respectively. Optical analyses of the two materials are conducted in the 0 to 14 eV energy range. The optical parameter study indicates that the materials are excellent in terms of photovoltaic and high-energy absorbent applications. The Seebeck coefficient shows that Pr_2_EuCoO_6_ and Pr_2_EuFeO_6_ are n- and p-type semiconductors, respectively. The elevated values of *ZT* at room temperature suggest that both materials have good thermoelectric efficiency, and the rising values of PF with increasing temperature suggest that the two materials can be used in high-temperature thermoelectric applications.

## Abstract 2

Spin-polarized DFT reveals stable half-metallic ferromagnetism and promising thermoelectric transport in Pr_2_EuMO_6_ (M = Co, Fe) double perovskites for spintronic and energy applications.

## Graphical abstract

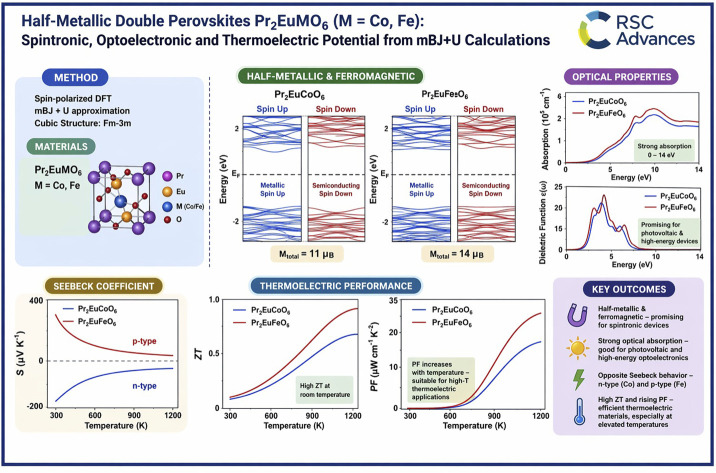

## 1 Introduction

The rising demand for innovative materials with enhanced features is propelling the technological development of the future. Consequently, the innovative design of new materials is changing considerably in the materials science field.^1^ Interest in the use of double perovskite materials has been both theoretical and empirical due to their numerous industrial and technical applications. The applications of these types of substances in spintronics, magneto-optics, half-metallic materials, thermoelectrics, and ferromagnetism are only a few of the areas that have found themselves in need of this flexibility. The materials also hold great potential in the production of semiconductors and magneto-dielectric materials; therefore, they rank among the top priorities of developing future electronics, energy conversion, and magnetic sensing technology.^2–4^ Rare earth (RE) elements and alkaline earth compounds have recently received significant attention because of their special properties and potential applications. The most important sub-type of double perovskites is the transition-metal perovskite.^5,6^ Considering that these substances can be tailored to work with the most recent technologies, such as electronics, magnetics, and energy-related uses, they are being carefully explored owing to their versatile array of possibilities.^4,7,8^

In the case of double perovskites, the general equation is A_2_BB′O_6_, which contains an oxygen atom O and the cations A, B, and B′. It is based on the fundamental perovskite chemical ABO_3_. Due to their ability to replace other components at the A, B and B′ positions, double perovskites have superior flexibility in the arrangement of atoms and thus are highly intriguing. These replacements can influence the optical, magnetic, and electrical properties of the material by enabling a very high level of control over the crystal structure. The tunability of double perovskites can be applied in many applications, including electrical devices, magnetism, spintronics, energy conversion, and storage.^7,9,10^ For decades, scientists have been studying these types of substances, focusing on their special qualities and uses, since the 1960s. Numerous investigations throughout the years have revealed their extraordinary flexibility and plasticity, igniting continued curiosity about theoretical and experimental studies.^11,12^ More than three hundred materials possessing a double perovskite structure have been effectively created. This illustrates the astounding adaptability of the double perovskite structure, which permits a wide range of structural and chemical changes. These substances are a major area of study in the field of materials science because they provide certain qualities for use in electronics, magnetism, and energy technologies.^13–15^

Due to their remarkable thermal properties, double perovskite (DPO) materials are also useful in thermal equipment, including sensors that monitor temperatures, coolers, motors, and thermocouples. Effective thermal control is essential in harsh settings, such as those encountered in airplanes or bases in space, where certain materials are especially well-suited for use. They are essential for space engineering and other cutting-edge thermoelectric systems because of their capacity to transform heat into electrical energy or to precisely regulate temperature.^16–18^ The addition of an electron's spin degree of motion has sparked an explosion in electronics and given rise to sophisticated spin-based technologies, or spintronics. The strong spin polarization of compounds is thought to be particularly beneficial for use in spintronic technologies. This novel method has several benefits over conventional electronic equipment, including higher processing rates, lower power usage, and permanent memory storage for information. Utilizing the inherent spin characteristics of electrons, spintronics holds promise for greatly enhancing device efficiency and opening the door for the future creation of electronic gadgets and devices. Further research on appropriate materials and procedures is essential to develop this innovative subject.^19,20^

A thorough literature review on double perovskites has been carried out to investigate previous studies on the materials. Towfiq *et al.*^21^ reported the half-metallic behavior and electronic structure of the Bi_2_FeMnO_6_ magnetic compound. Belhachi *et al.*^6^ also reported the half-metallic quality and electronic, magnetic and optical properties of the Ba_2_CoRhO_6_ compound. Mudasir *et al.*^22^ reported the spin-polarized properties of double perovskite K_2_GeMnX_6_ (X = Cl, Br, I). Bhuyan *et al.*^23^ reported the spintronic properties of Nd_2_CrFeO_6_, a double perovskite. Caid *et al.*^24^ explored the spin-dependent physical properties of Cs_2_B′GeCl_6_ (B′ = Zn, Cd), a double perovskite family. A Ali *et al.*^25^ recently investigated the magnetic, thermoelectric, and optoelectronic properties of Ba_2_GdXO_6_ (X = Nb and U) double perovskites using DFT analysis. These materials are ferromagnetic and semiconducting and exhibit excellent optical and thermoelectric characteristics. A Ali *et al.*^26^ reported double perovskite materials owing to their optoelectronic and magnetic properties using a spin-based first-principles investigation. These investigations suggest the antiferromagnetic and half-metallic nature of Sr_2_UFeO_6_ and the ferromagnetic and semiconducting nature of Sr_2_UNiO_6_. Zia *et al.*^27^ performed spin-based computations of Cs_2_XMoBr_6_ (X = Na, Li) double halide perovskites for investigations of the magnetic, thermoelectric, and optoelectronic characteristics of materials. Mazumdar *et al.*^28^ experimentally synthesized Pr_2_FeCrO_6_, a double perovskite material, and investigated its magnetic, structural, and magneto-caloric properties. Dhilip *et al.*^29^ computationally and experimentally analyzed the Pr_2_CoFeO_6_ double perovskite and investigated the structural, optoelectronic, and magnetic characteristics of the material. Saadi^30^ elaborated the half-metallic and magnetic nature of Sr_2_GdReO_6_ material using first principles investigations. The material is found to be half-metallic and ferromagnetic in nature. The ferromagnetic and half-metallic nature of X_2_MnUO_6_ (X = Sr or Ba) materials has been revealed by a first principles study using the FP-LAPW method.^31^ Saadi *et al.*^32^ reported the ferromagnetic and half-metallic nature of RBaMn_2_O_6−*δ*_ (X = Nd, Pr, La and *δ* = 0, 1) compounds using a DFT study. A first principles investigation was performed to predict the thermoelectric and half-metallic characteristics of Sr_2_EuReO_6,_ a double perovskite compound.^33^

The present research focuses on the first principles investigations of novel Pr_2_EuMO_6_ (M = Co, Fe) double oxide perovskites. This study aims to examine the structural, electronic, magnetic, and thermoelectric characteristics of double perovskites Pr_2_EuMO_6_ (M = Co, Fe) using first-principles calculations. The primary goals of this study are to investigate how the selection of transition metal (Co *vs.* Fe) affects the electronic band structure and magnetic ordering and to assess whether these materials display half-metallic ferromagnetism, positioning them as potential candidates for spintronic applications. This study also examines the thermoelectric performance of both compounds in relation to temperature, aiming to evaluate their potential for energy conversion applications. Variations in transition metals are believed to significantly influence their physical properties, resulting in unique magnetic moments and thermoelectric behaviors across various temperature ranges. The detailed research literature review predicts, both theoretically and experimentally, the novelty of the materials under study.

## 2 Computational method

The FP-LAPW technique, which has been implemented inside the DFT-based WIEN2K computational framework,^34^ is utilized to perform spin-polarized computational tasks to investigate the magnetic electronic and optical properties of materials in the cubic (*Fm*3̄*m*) space group.^35,36^ The WEIN2K code is selected to perform the structural, electronic, optical, and magnetic calculations of the materials with high accuracy, as it is based on the full potential method, which precisely incorporates the effects of core electrons, providing benefits over other DFT-based codes that utilize pseudopotentials. To account for both the precise band gap estimation and strong on-site Coulomb interactions in the localized d/f orbitals, electronic structure calculations were carried out using the modified Becke–Johnson (mBj) exchange potential in combination with the Hubbard U correction (mBj + U).^37,38^ The effective Hubbard U values are 6.0 eV for Pr-4f,^39^ 6.5 eV for Eu-4f,^40^ 3.5 eV for Co-3d,^41^ and 4.5 eV for Fe-3d orbitals.^42^ Although DFT + U, along with the TB-mBJ method, was used in the current study to consider the strong electron correlation in transition metal sites, it is acknowledged that the approach could still have a self-interaction error (SIE), which could negatively impact the accuracy of electronic property predictions. Other methods, including hybrid functionals, self-interaction-corrected (SIC) methods and other methods, have been demonstrated to provide better accuracy than standard DFT + U for systems containing transition metals.^43,44^ The wave function has been extended utilizing the sphere-shaped harmonic basis set as much as *l*_max_ = 10 across the not interconnected muffin-tin spheres. A plane-wave basis with a threshold value of *R*_MT_*K*_max_ = 7 was implemented to extend the wave function throughout the interstitial region. The cut-off energy is chosen as −6.0 Ry to distinguish between the core and valence states. The incorporation of the Brillouin zone has been completed by employing a 10 × 10 × 10 *k*-mesh Monkhorst-Pack.^45,46^ In the present calculations, the energy and convergence criteria are considered whenever the charge difference is smaller than 0.001 e a.u.^−3^ per unit cell and the overall energy is steady within 0.0001 Ry. To avoid spurious oscillations and achieve higher quantitative accuracy, the optical characteristics were calculated using 15 × 15 × 15 *k*-points in the first Brillouin zone.^47–50^ To look into optical characteristics, the material's complex dielectric function is computed, as follows:^51^

1 *ε*(*ω*) = *ε*_1_(*ω*) + *ε*_2_(*ω*).

The Kramers–Kronig relations were used to obtain the real component *ε*_1_ and the imaginary part *ε*_2_, which were determined by adding a suitably high number of unfilled states.^52^ Further, both *ε*_1_ and *ε*_2_ can be expressed mathematically using the following two equations:2
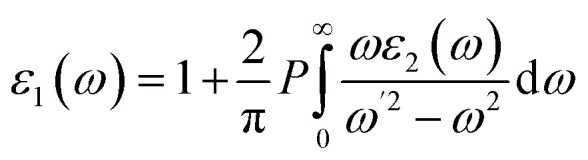
3
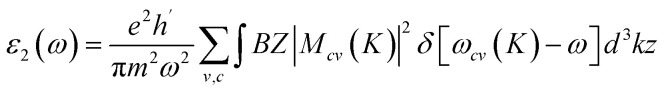


The real and imaginary parts of the dielectric function are utilized to determine the material's additional optical characteristics, employing the following derived formulas:4
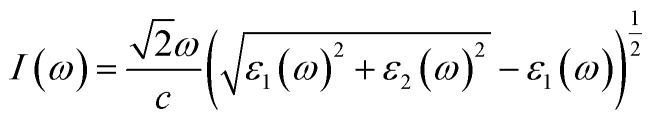
5
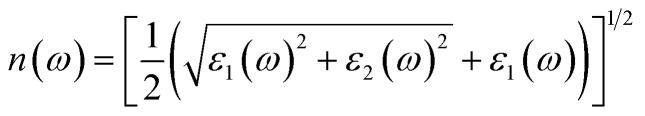
6
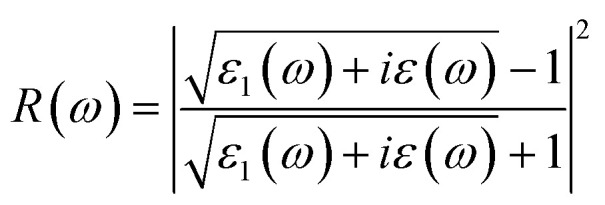
7
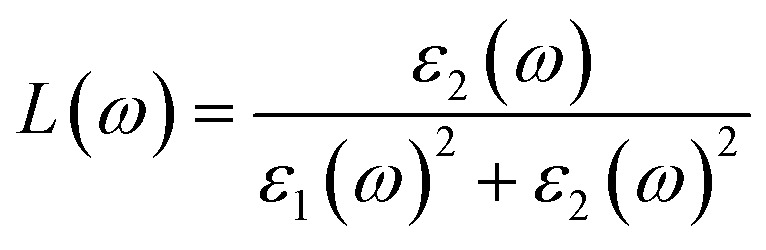


To compute the thermoelectric properties, the semi-classical Boltzmann transport theory was applied using the BoltzTraP code.^53^ The Seebeck coefficient, electrical conductivity and electronic thermal conductivity were determined using this code. The thermoelectric transport properties were calculated using the semi-classical Boltzmann transport theory within the constant relaxation time approximation (CRTA), as implemented in the BoltzTraP code. In this approach, only the electronic contribution to the transport coefficients (Seebeck coefficient, electrical conductivity, and electronic thermal conductivity) is evaluated based on the calculated electronic band structure. The lattice thermal conductivity (*κ*_l_) is not included in this framework, as its calculation requires anharmonic phonon interactions and higher-order force constants, which are beyond the scope of the present study. In order to obtain precise values, a 1000 (10 × 10 × 10) *k*-mesh is employed to integrate the electronic energy levels of our mBJ + U calculation.

## 3 Results and discussions

### 3.1 Structural properties

**Fig. 1:**
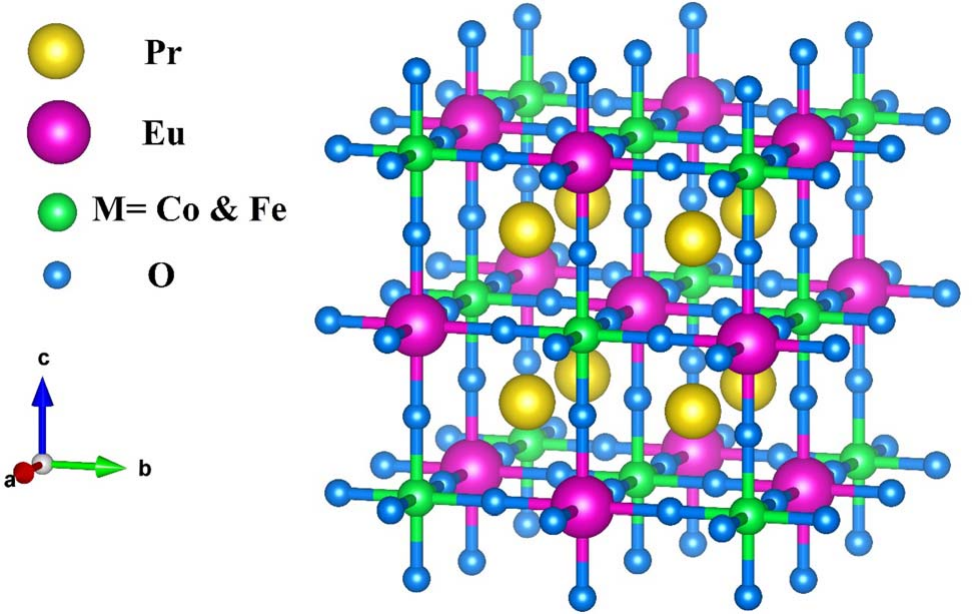
Crystallographic structures of the Pr_2_EuMO_6_ (M = Co, Fe) perovskite materials.

Both Pr_2_EuCoO_6_ and Pr_2_EuFeO_6_ materials crystallize in the cubic *Fm*3̄*m* space group, as displayed in Fig. 1. The structures of these materials are optimized to obtain optimized bond lengths, lattice contents, and volumes. The energy *vs.* volume optimization curves are depicted in Fig. 7(a and b). The nature of the bonds among the material elements can be determined by computing the electronegativity difference between them. The electronegativity difference can be determined using Pauling values for each constituent element.^54,55^ For Pr_2_EuCoO_6_ and Pr_2_EuFeO_6_, the electronegativity difference for Pr–O, Eu–O, Co–O, and Fe–O bonds is 2.31, 2.24, 1.56, and 1.61, respectively, confirming that Pr–O and Eu–O have an ionic bonding nature while Co–O and Fe–O have a polar covalent bonding nature.^56^ The bonding nature can further be confirmed by studying the DOS plots (see Fig. 4), which show negligible hybridization between Pr–O and Eu–O, indicating ionic and strong hybridization near the Fermi level in the up-spin configurations between Co–O and Fe–O in Pr_2_EuCoO_6_ and Pr_2_EuFeO_6_, respectively, suggesting the covalent character of the materials. The tolerance factor provides insights into the crystal structure phase stability of the material. The concept of the tolerance factor for phase stability was first introduced by Megaw.^57^ Furthermore, Goldschmidt introduced the modified form of the tolerance factor, which is based on the ionic radii of the elements of the double perovskite materials. The modified form of the Goldschmidt tolerance factor (*τ*) formula for the materials under study is as follows:^58^8
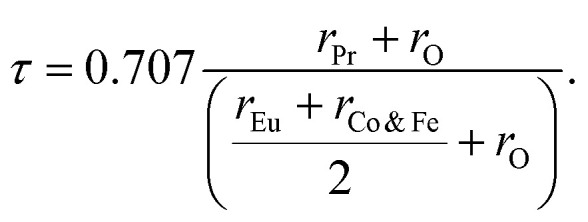
Here, the *r*_Pr_, *r*_Eu_, *r*_Co_, *r*_Fe_, and *r*_O_ are the ionic radii of the elements Pr, Eu, Co, Fe, and O, respectively. The values of these ionic radii are suggested by Shannon *et al.*^59^ The radii (in Å) of the material elements are *r*_Pr_ = 1.126, *r*_Eu_ = 1.120, *r*_Co_ = 0.545, *r*_Fe_ = 0.55, and *r*_O_ = 1.35, respectively. The computed tolerance factors of the Pr_2_EuCoO_6_ and Pr_2_EuFeO_6_ materials are 0.923 and 0.920, respectively. The value range of the tolerance factor for materials stable in the cubic (*Fm*3̄*m*) space group is 0.9–1, which suggests that the computed tolerance of the materials lying in the stable crystallographic phase range confirms the stable crystallographic phase of the materials.^60^ The prediction of formation energy helps in understanding the thermodynamic stability of materials.^61,62^ The formation energy Δ*H*_F_ of the Pr_2_EuMO_6_ (M = Co, Fe) materials can be computed by employing the following relation:9
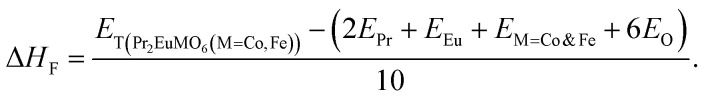


The relation contains the total energy of the material, *E*_T_, and the energies of the constituent elements *E*_Pr_, *E*_Eu_, *E*_M = Co & Fe_ and *E*_O_ for Pr, Eu, (M = Co & Fe) and O elements, respectively. These energies are obtained by performing energy *vs.* volume optimizations of both materials in the most stable magnetic phase (FM). The computed formation energies per atom (in eV) of the materials Pr_2_EuCoO_6_ and Pr_2_EuFeO_6_ are −7.42 and −3.37, respectively. The negative formation enthalpy indicates the thermodynamic stability of the materials under study.^25^

#### 3.1.1 Phonon stability

**Fig. 2:**
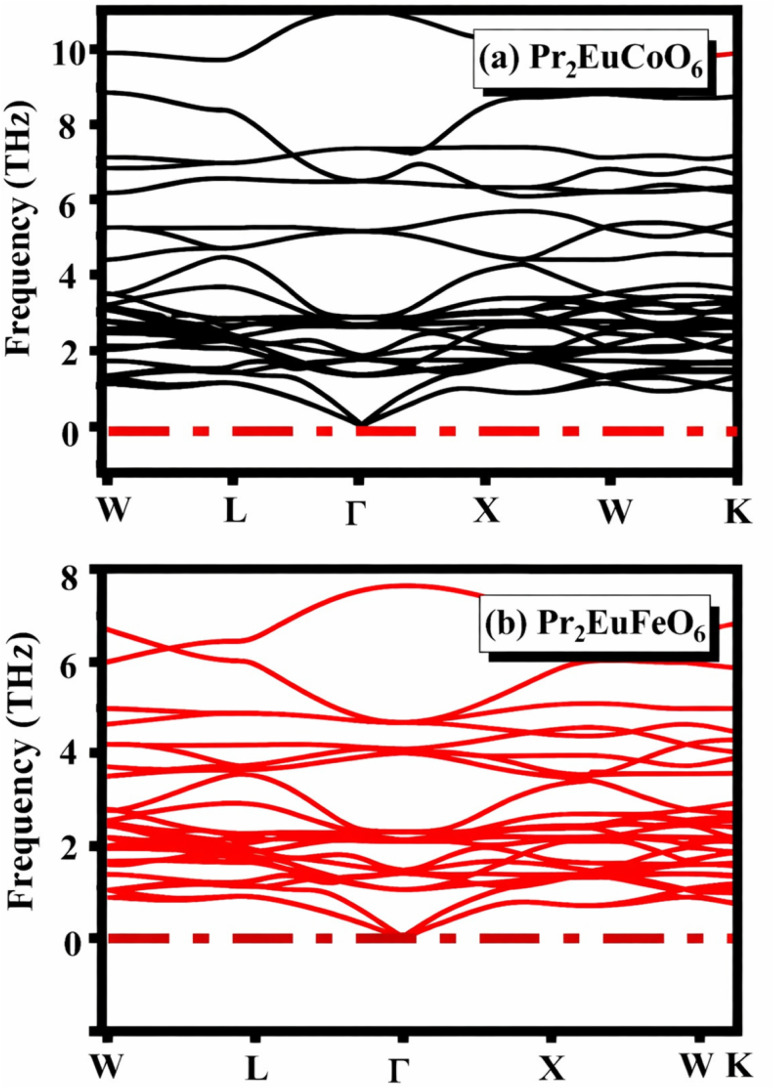
Phonon dispersion relations of (a) Pr_2_EuCoO_6_ and (b) Pr_2_EuFeO_6_ along the high-symmetry path, *W*–*L*–*Γ*–*X*–*W*–*K*, in the Brillouin zone. The absence of imaginary frequencies (indicated by the dashed horizontal line at 0 THz) confirms the dynamical stability of both compounds. The phonon branches are shown in black for Pr_2_EuCoO_6_ and in red for Pr_2_EuFeO_6_.

The phonon dispersion curves of Pr_2_EuCoO_6_ and Pr_2_EuFeO_6_, shown in Fig. 2(a) and (b), respectively, provide direct insight into their dynamical stability. In both compounds, all phonon branches remain positive throughout the high-symmetry Brillouin zone path (*W*–*L*–*Γ*–*X*–*W*–*K*), with no imaginary frequencies observed. This absence of soft modes indicates that both structures are dynamically stable in their optimized configurations. Notably, the acoustic branches smoothly approach zero frequency at the *Γ*-point, as expected from translational invariance, further confirming the numerical robustness of the calculations.

A comparison between the two systems reveals subtle differences in the optical phonon region. Pr_2_EuCoO_6_ exhibits a broader distribution of high-frequency modes extending beyond ∼10 THz, while Pr_2_EuFeO_6_ shows a relatively compressed spectrum with maximum frequencies below ∼8 THz. This shift can be attributed to differences in the mass and bonding characteristics of Co–O and Fe–O octahedra, where stronger bonding interactions in the Co-based system lead to higher vibrational frequencies.

### 3.2 Electronic properties

**Fig. 3:**
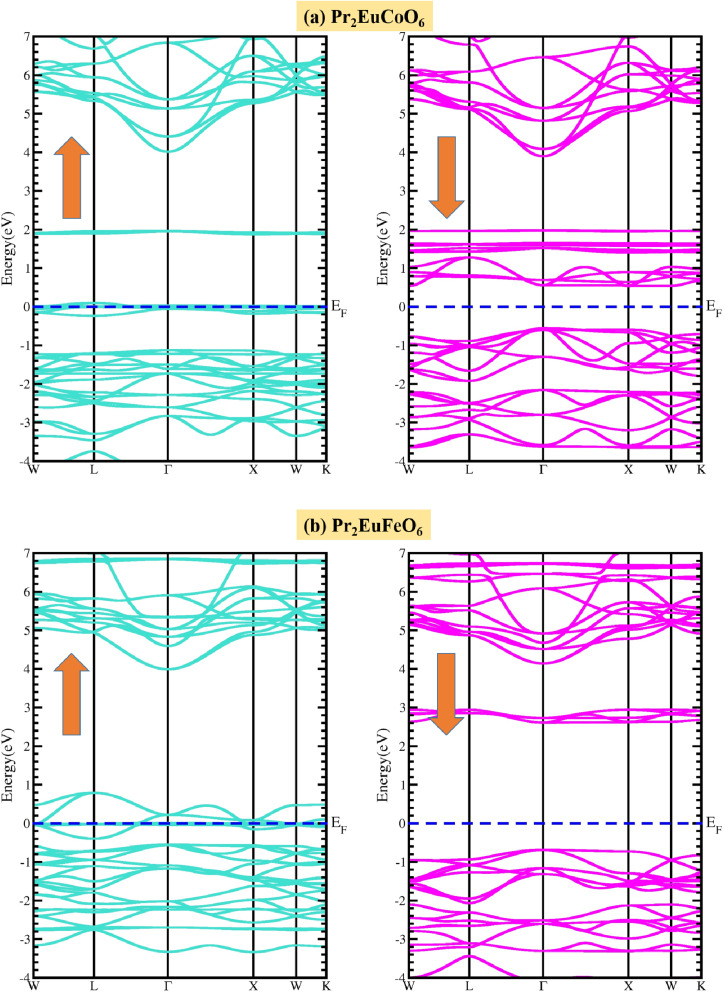
Spin-polarized electronic band structures of the (a) Pr_2_EuCoO_6_ and (b) Pr_2_EuFeO_6_ perovskites.

**Fig. 4:**
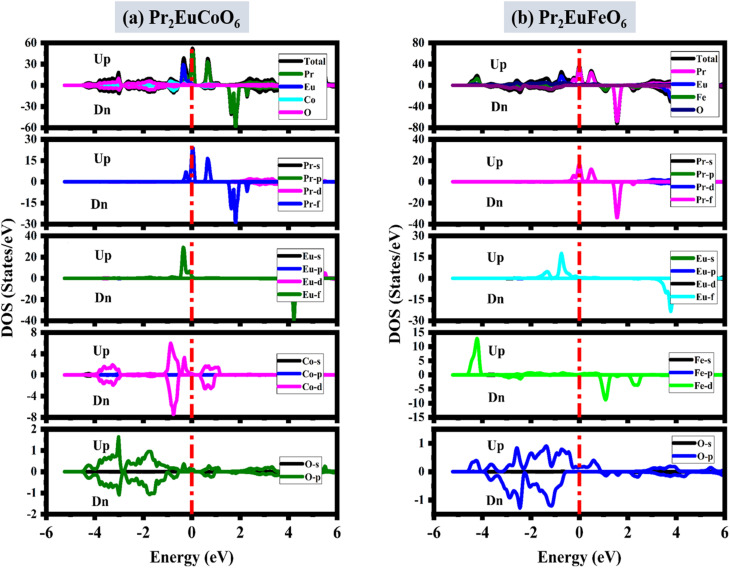
Spin-polarized total and partial density of states of the (a) Pr_2_EuCoO_6_ and (b) Pr_2_EuFeO_6_ perovskites.

The cubic phase of the double perovskite oxide (DPO) material Pr_2_EuMO_6_ (M = Co and Fe) and its magneto-electronic characteristics have been studied. The spin-polarized band structures (BSs) of Pr_2_EuCoO_6_ and Pr_2_EuFeO_6_ are calculated within the energy spectrum of −4 to 4 eV on the *Y*-axis *versus* the high symmetric directions *W*, *L*, *Γ*, *X*, *W*, and *K* of the first Brillouin zone, as depicted in Fig. 3. According to the BS plots, both compounds have identical electronic band topologies, suggesting the same electronic activity within the studied energy spectrum. The plots suggest that both materials, Pr_2_EuCoO_6_ and Pr_2_EuFeO_6_, exhibit metallic characteristics in up-spin configurations, as the valence band (VB) energy levels cross the Fermi level, facilitating free electron motion. On the other hand, the down spin state of both materials demonstrates semiconducting behavior, with the conduction band minimum (CBM) and valence band maximum (VBM) prominently distinguished by forbidden energy gaps. Near the *X* symmetrical path, this shortest partition generates a straight band gap, highlighting the compound's versatility for spin-dependent purposes. The determined energy band gap values in spin down for Pr_2_EuCoO_6_ and Pr_2_EuFeO_6_ are 1.2 eV and 3.0 eV, respectively. The band gaps indicate their appropriateness for usage, whereby adjustable electrical characteristics are beneficial. These materials with a metallic nature in the spin-up and semiconducting in the spin-down channels are known to be half-metallic.^63^ The BS of both materials suggests that the overall nature of these materials is half-metallic. Half-metallic materials have attracted considerable interest in recent years because of their distinctive electronic structure, displaying metallic characteristics in one spin direction while exhibiting semiconducting properties in the other.^64,65^ Their ability to produce a fully spin-polarized current makes them perfect for use in spintronic applications. The distinct characteristics of half-metals have prompted studies into their magnetoresistance and possible uses in electronic devices.^66^ The contribution of material elements and electronic states to the conductivity and electronic nature of materials is explained by the density of states (DOS) analysis. The elemental contributions are given by the total density of states (TDOS), while the electronic state contribution is given by the partial density of states (PDOS), as displayed in Fig. 4(a and b). The DOS analysis of both materials provides more insight into the magnetic and conducting characteristics of the materials by highlighting their unique spin-polarized electronic structures. The plots suggest that the valence band in the up spin is formed from 0 to −5 eV, and the conduction band from 0.5 to 6 eV energy ranges. Additionally, in the down spin configurations, the valence band of Pr_2_EuCoO_6_ (see Fig. 4(a)) is from −0.5 to −5 eV, the conduction band is from 1.5 to 6 eV, the valence band of Pr_2_EuFeO_6_ (see Fig. 4(b)) is from 0.8 to 5 eV, and the conduction band ranges from 1 eV to 6 eV. The DOS plots follow the energy band gap explanations of materials by electronic structure analysis. The electronic structures in Pr_2_EuCoO_6_ and Pr_2_EuFeO_6_, which are mostly provided by the states of the Pr element, and the PDOS indicate that the Pr-f state overlaps the Fermi level in up spin configurations, which confirms the metallic nature of the materials. The valence band maximum and conduction band minimum of Pr_2_EuCoO_6_ and Pr_2_EuFeO_6_ in the up spin are made up of the Pr-f state. Similarly, in the down spin configuration, the valence band maximum and conduction band minimum of Pr_2_EuCoO_6_ material are formed by the Co-d state; the valence band maximum of Pr_2_EuFeO_6_ is developed by the O-p state, and the conduction band minimum is due to Fe-d states. The half-metallic nature of the materials is confirmed by the DOS plots. The DOS graphs additionally demonstrate significant hybridization involving the transition metals of Pr_2_EuCoO_6_, including the hybridization among Eu-f and Co-d (in up-spins) and Co-d and O-p states (in down-spins). The hybridizations also take place between the Eu-f and O-p states (in up-spins) of the Pr_2_EuFeO_6_ material. Both Pr_2_EuCoO_6_ and Pr_2_EuFeO_6_ exhibit half-metallic properties, attaining 100% spin polarization, resulting from the strong metallicity in the majority spin channel and the semiconducting function in the minority spin channel. This special characteristic raises the possibility that these materials might be used as efficient spin injectors in spintronic products, whereby information transportation and storage depend on the ability to manipulate the electron spin.

### 3.3 Optical properties

**Fig. 5:**
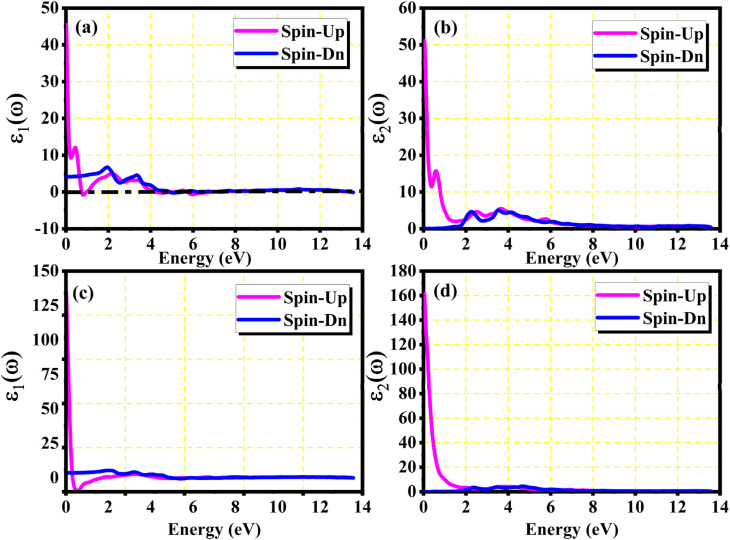
Real *ε*_1_(*ω*) and imaginary *ε*_2_(*ω*) parts of the dielectric function *ε*(*ω*) of (a and b) Pr_2_EuCoO_6_ and (c and d) Pr_2_EuFeO_6_ in both spin configurations.

A useful technique for figuring out a material's band structures and the response of the materials to the incident light is the analysis of the optical properties. The most significant aspect of the solid crystal's optical characteristics is its complex dielectric function, which is closely connected to the BS of crystals and consists of two parts: real and imaginary parts. The optical characteristics parameters are investigated in the photon energy range of 0–14 eV to study optical transitions. The computed real and imaginary parts of the materials in both spin configurations are displayed in Fig. 5(a–d).

**Fig. 6:**
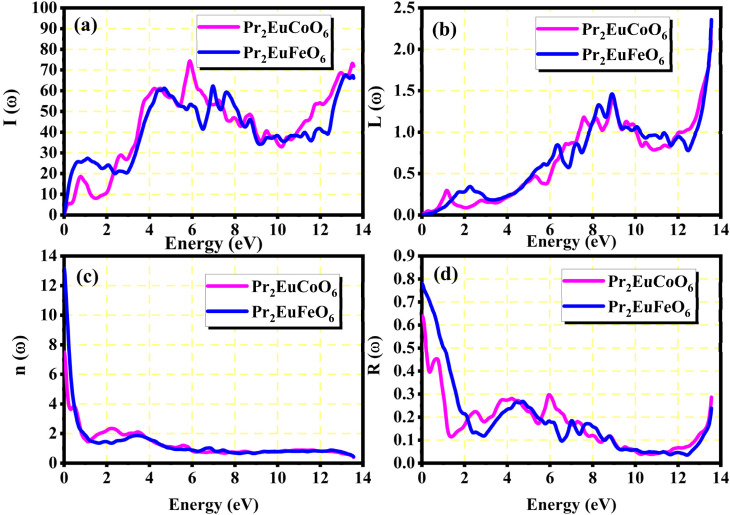
(a) Absorption coefficient *I*(*ω*), (b) energy loss *L*(*ω*), (c) refractive index *n*(*ω*), and (d) reflectivity spectra *R*(*ω*) of the Pr_2_EuMO_6_ (M = Co and Fe) perovskites.

The real parts indicate the polarization of materials in response to incident electromagnetic radiation. This also indicates energy storage by materials in electric fields.^67^ The value of *ε*_1_(*ω*) at 0 eV, which is known as the static value *ε*_1_(0), is depicted in Fig. 5(a); for Pr_2_EuCoO_6_, the *ε*_1_(0) values are 45.0 (up-spin) and 4.5 (down-spin) states, while for Pr_2_EuFeO_6_, as illustrated in Fig. 5(c), these values are 137.5 (up-spin) and 12.0 (down-spin) states. The higher values in the up-spin configurations indicate and confirm the metallic nature, and the small values represent the semiconducting behavior of the materials, as depicted in the band structures (BSs) of the materials (see Fig. 3). This also confirms the spin-polarized electronic nature (half-metallic) of the materials. The *ε*_1_(0) higher value of Pr_2_EuFeO_6_ than Pr_2_EuCoO_6_ also indicates stronger hybridization of the Fe-d and O-p states than the Co-d and O-p states. The highest values of *ε*_1_(*ω*) for Pr_2_EuCoO_6_ are located in the visible region of the optical spectrum at energies of 0.42 eV (up spin) and 1.9 eV (down spin), with negligible values in the higher optical regions. The imaginary part *ε*_2_(*ω*) of the dielectric function, depicted in Fig. 5(b and d), expresses energy absorption or dissipation by the materials. The peaks in the imaginary parts express the intra-band electronic transitions. The plot suggests that the *ε*_2_(*ω*) values for Pr_2_EuCoO_6_, depicted in Fig. 5(b), reach their highest values at low energies in the up spin configurations and reach a threshold of 1.78 eV in the down spin configurations. The values of *ε*_2_(*ω*) for Pr_2_EuFeO_6_, shown in Fig. 5(d), reach their highest values at low energies in the up spin configurations and reach a threshold of 1.1 eV in the down spin configurations. The plots indicate certain absorptions at 24 eV for both materials and suggest negligible absorption at higher energies. The highest value of *ε*_2_(*ω*) at low energies indicates the metallic nature in the up spin, and threshold values in the down spin indicate the semiconducting nature of the materials, supporting the half-metallic electronic nature of the materials.^68^ The other computed optical parameters, including the absorption, energy loss, refractive index, and reflectivity of both materials, in an up-spin configuration, are displayed only in Fig. 6(a–d). The absorption coefficient (*I*(*ω*)) directly quantifies the optical power absorbed by the material. A high *I*(*ω*) at a specific photon energy signifies an intense interaction with light, along with effective energy dissipation or conversion within that spectral range. The absorption of energy by the materials can be understood by studying the absorption coefficient of the materials, as depicted in Fig. 6(a). The absorption coefficient *I*(*ω*) is a crucial metric for characterizing any optoelectronic compound.^69,70^ The absorption of the materials indicates that the absorption of incident radiation starts from the visible region, increases with an increase in incident radiation energy and reaches its maximum value at 21.8 eV for Pr_2_EuCoO_6_ and 22.5 eV for Pr_2_EuFeO_6_ material. All the absorptive peaks indicate intra-band electronic transitions. The maximum absorptive peak for both materials indicates the electronic transition from the Eu-f to Pr-f states. *L*(*ω*) represents the energy loss function, an important parameter that demonstrates the loss of energy from materials, as displayed in Fig. 6(b). The results depict that the energy loss by the materials is small in the visible energy range of the spectrum, increases with the increase in incident radiation energy and reaches the highest value at 22.7 eV energy. The refractive index *n*(*ω*) explains the dispersive nature of the materials and is plotted in Fig. 6(c). The static *n*(0) values are 13 for Pr_2_EuFeO_6_ and 7.5 for Pr_2_EuCoO_6_. The maximum dispersion of the incident radiation takes place in the visible region. The surface morphology can be explained by the study of the reflectivity coefficient of the materials, as displayed in Fig. 6(d). The static reflectivity *R*(0) has values of 0.65 (65%) and 0.78 (78%) for Pr_2_EuCoO_6_ and Pr_2_EuFeO_6_, respectively. The higher percentage of reflectivity under low static conditions in up spin confirms the metallic nature of the materials. The maximum reflectivity peaks indicate the plasmon resonance in the specified energy ranges of the spectrum. The optical parameters and electronic nature of the materials suggest that they are promising candidates for spintronic, photovoltaic, and solar cell applications.

### 3.4 Magnetic nature

**Fig. 7:**
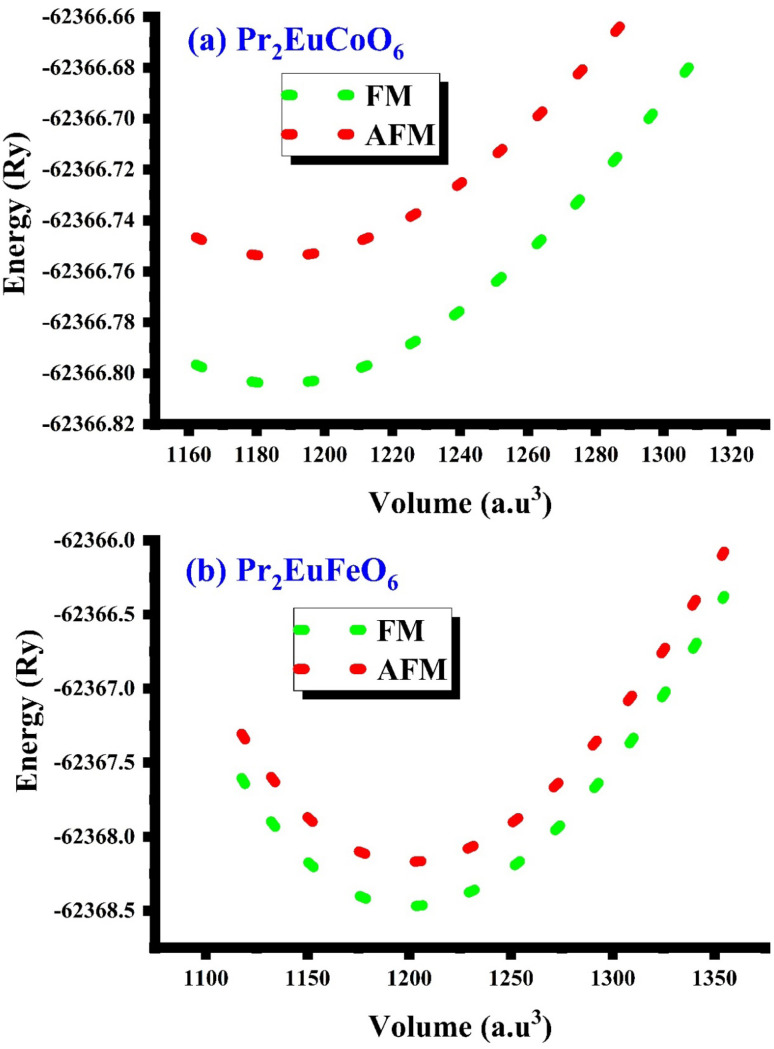
Optimization curves in the ferromagnetic (FM) and anti-ferromagnetic (AFM) magnetic phases of the (a) Pr_2_EuCoO_6_ and (b) Pr_2_EuFeO_6_ perovskites.

The magnetic properties of these double perovskites are crucial for magneto-optic applications, data storage systems, and spintronics because of their many magnetic properties, including ferromagnetism, ferrimagnetism, and anti-ferromagnetism.^71^ Materials with transitions and lanthanide constituent elements, having unpaired electrons in the d or f orbital, show magnetic behavior.^72,73^ The unpaired electrons in the outermost orbit of the material's element create magnetic moments that lead to the magnetic behavior of the material. To understand the magnetic strength of the Pr_2_EuMO_6_ (M = Co and Fe) materials, first-principles spin-based computations are performed. The computed magnetic moments (in *µ*_B_) of the materials under study are displayed in Table 1. The table displays the magnetic moment due to interstitial sites (MM_int_), due to Pr (MM_Pr_), due to Eu (MM_Eu_), due to M = Co and Fe (MM_M_), and due to O (MM_O_), and the total magnetic moment of the material is (MM_total_). The total magnetic moments of the Pr_2_EuCoO_6_ and Pr_2_EuFeO_6_ are 11 (*µ*_B_) and 14 (*µ*_B_), respectively. The integral value of the total magnetic moments indicates the ferromagnetic nature of the materials.^74^ The larger value of the total magnetic moment of Pr_2_EuFeO_6_ indicates strong coupling between Eu and Fe compared to Eu and Co in the Pr_2_EuCoO_6_ material. The stronger coupling of Fe compared to Co suggests a stronger intrinsic magnetic moment of Fe than of Co. The table displays that the major contribution to the total magnetic moment of both materials is due to unpaired electrons of Pr, Eu, and Fe (only for Pr_2_EuFeO_6_), while other elements, including Co (for Pr_2_EuCoO_6_), have negligible contributions. The small contribution of the Co magnetic moment may be due to the lower spin state adopted by the unpaired electrons of the element. The negative magnetic moment of Co indicates antiferromagnetic alignment with the Pr and Eu spin configurations. Antiparallel alignment may arise from super-exchange interactions facilitated by oxygen atoms within the perovskite structure. The parallel alignment of Fe with Pr and Eu atom spins in Pr_2_EuFeO_6_ causes a higher value of the total magnetic moment than the Pr_2_EuCoO_6_ material's total magnetic moment. Furthermore, the magnetic nature of the materials can be confirmed using volume optimization analysis in different magnetic phases, including ferromagnetic (FM) and anti-ferromagnetic (AFM) phases. The computed optimization curves of both materials in the magnetic phases are plotted in Fig. 7(a and b). The optimization plots demonstrate that the ferromagnetic phase of both materials is the most stable magnetic phase among the other phases, which predicts the ferromagnetic nature of both materials. Table 2 illustrates the analytical analysis of the FM and AFM stability margin Δ*E* (in Ry and meV). The positive values of Δ*E* indicate and confirm the FM nature of the materials. Ferromagnetic materials have a large number of potential applications. Ferromagnetic materials offer a wide range of uses across different industries. They are utilized in magnetic data storage, transformer cores, electrical generators, and permanent magnets.^75^ In technologies such as data storage systems, refrigeration, and medical research, these materials are useful because they display special characteristics when subjected to external magnetic fields.^76^ New developments in ferromagnetic materials have expanded the potential uses of magneto-optical, magneto-resistive, magnetostrictive, and nanocrystalline soft magnetic materials.^75^

**Table 1:** Magnetic moments (*µ*_B_) of the Pr_2_EuMO_6_ (M = Co and Fe) materials

Materials	MM_int_ (*µ*_B_)	MM_Pr_ (*µ*_B_)	MM_Eu_ (*µ*_B_)	MM_M_ (*µ*_B_)	MM_O_ (*µ*_B_)	MM_total_ (*µ*_B_)
Pr_2_EuCoO_6_	0.30606	2.42867	6.24646	−0.04431	−0.06980	11
Pr_2_EuFeO_6_	0.50719	2.26215	6.10179	4.05157	−0.20723	14

**Table 2:** Magnetic ground state verification: total energy and stability margin Δ*E* (in Ry and meV) for the FM and AFM phases of the Pr_2_EuMO_6_ (M = Co and Fe) materials

Materials	*E*_FM_ (Ry)	*E*_AFM_ (Ry)	Δ*E* (Ry) = *E*_FM_ − *E*_AFM_	Δ*E* (meV) = *E*_FM_ − *E*_AFM_	Magnetic phase
Pr_2_EuCoO_6_	−62 366.804	−62 366.752	0.052	+707.49	FM
Pr_2_EuFeO_6_	−62 368.460	−62 368.170	0.290	+3945.65	FM

### 3.5 Thermoelectric properties

**Fig. 8:**
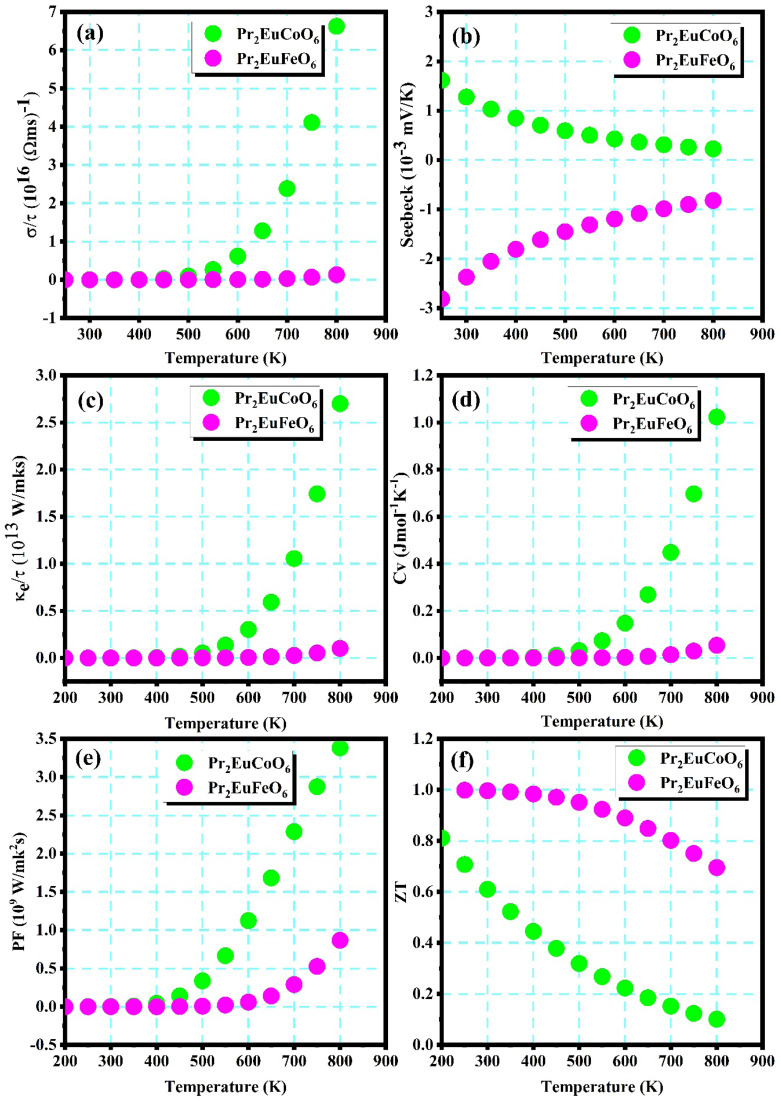
Thermoelectric parameters of Pr_2_EuMO_6_ (M = Co and Fe): (a) electrical conductivity (*σ*/*τ*), (b) Seebeck coefficient (*S*), (c) electronic thermal conductivity (*κ*_e_), (d) specific heat capacity at constant volume *C*_v_, (e) power factor (PF) and (f) *ZT*.

The substantial rise in fossil fuel consumption due to recent global economic expansion has led to severe energy crises and considerable degradation of the environment. Excess heat can be transformed into electricity utilizing thermoelectric materials to mitigate energy crises and diminish hazardous emissions. The favorable thermoelectric properties of perovskite compounds, namely their elevated Seebeck coefficient and high carrier concentration, have garnered significant interest.^77^ Thermoelectric parameters (in the spin-down channel), including electrical conductivity, Seebeck coefficients, electronic thermal conductivity, specific heat capacity *C*_v_, the power factor (PF), and *ZT*, are computed using the BoltzTraP computational code. The thermoelectric parameters are displayed in Fig. 8(a–f). The electrical conductivities of both materials are computed from 200 to 900 K, as displayed in Fig. 8(a), which shows the conductivity of electrons by the materials caused by the applied temperature gradient. The plots demonstrate the increasing trend of the electrical conductivity of the materials with temperature, which is a characteristic of semiconducting materials whose carrier mobility increases with temperature. Pr_2_EuFeO_6_ exhibits smaller electrical conductivity compared to Pr_2_EuCoO_6_ across the temperature range. The electrical conductivity of Pr_2_EuFeO_6_ increases slightly beyond 800 K, while Pr_2_EuCoO_6_ has an increasing electrical conductivity beyond 450 K of temperature. The electrical conductivity is strongly influenced by the energy band gaps of the materials; Pr_2_EuCoO_6_ has a smaller energy band gap (see Fig. 3(a and b)) compared to Pr_2_EuFeO_6_, resulting in higher electrical conductivity for Pr_2_EuCoO_6_ across the temperature range. The Seebeck coefficient is an important parameter that measures the voltage generated across the ends of materials by employing a temperature gradient across the ends. The computed Seebeck coefficient (*S*) for both materials is shown in Fig. 8(b). The plots display different behaviors for both materials with an increase in temperature. The Seebeck coefficient of Pr_2_EuFeO_6_ is −2.7 mV K^−1^ at 200 K and increases gradually to −0.8 mV K^−1^ at 800 K, while that of Pr_2_EuCoO_6_ is 1.7 mV K^−1^ at 200 K and decreases gradually to 0.20 mV K^−1^ at 800 K. In semiconducting materials, the Seebeck coefficient is an essential indicator of the type of carrier. The decreasing trend in the Seebeck coefficient of Pr_2_EuCoO_6_ indicates the n-type, and the increasing trend of Pr_2_EuFeO_6_ indicates the p-type nature of the materials.^78^ The thermal conductivity of the materials is determined by lattice vibrations and electronic movements. The present study focuses on electronic thermal conductivity, as the computation of lattice thermal conductivity requires more computational resources. The electronic thermoelectric conductivity of the materials is displayed in Fig. 8(c) and computed from the 200–900 K temperature range. Electronic thermal conductivity demonstrates the heat flow through materials *via* free electrons or holes. The thermal conductivity for both materials shows a similar trend of rise with temperature. The thermal conductivities of Pr_2_EuCoO_6_ and Pr_2_EuFeO_6_ start to increase beyond 450 K and 600 K, respectively. The thermal conductivity of Pr_2_EuCoO_6_ is significantly higher than that of Pr_2_EuFeO_6_ across the temperature range. The thermal conductivity of Pr_2_EuCoO_6_ is 0.21 × 10^13^ W (mKs)^−1^ at 450 K and rises to 2.75 × 10^13^ W (mKs)^−1^ at 800 K. Similarly, the thermal conductivity for Pr_2_EuCoO_6_ is 0.21 × 10^13^ W (mKs)^−1^ at 600 K and 0.3 × 10^13^ W (mKs)^−1^ at 800 K. The higher electronic thermal conductivity of Pr_2_EuCoO_6_ compared to that of Pr_2_EuFeO_6_ is due to the higher charge carrier concentration of Pr_2_EuCoO_6_. The specific heat capacity *C*_v_ at a constant volume for both materials, displayed in Fig. 8(d), has a similar nature of variation with temperature rise, which is the measure of heat required to increase the temperature of a material by 1 K. The specific heat capacity *C*_v_, though thermodynamic, is included here due to its role in evaluating lattice thermal conductivity, which directly affects thermoelectric performance. The specific heat capacity *C*_v_ for Pr_2_EuCoO_6_ is very small up to 450 K and increases to 1.02 J mol^−1^ K^−1^ at 800 K, and that for Pr_2_EuFeO_6_ also has a small value up to 650 K and rises to 0.05 J mol^−1^ K^−1^ at 800 K. The maximum value of *C*_v_ for Pr_2_EuCoO_6_ is higher than that of Pr_2_EuFeO_6_. The elevated maximum *C*_v_ of Pr_2_EuCoO_6_ may be ascribed to its greater atomic mass, distinct bonding properties, or expanded vibrational spectrum. The performance of thermoelectric materials is commonly assessed by their power factor (PF), defined as the product of the square of the Seebeck coefficient (*S*) and electrical conductivity (*σ*).^79^Fig. 8(e) displays the computed power factor (PF) of both materials. The plots indicate that the PF of Pr_2_EuCoO_6_ is higher than that of Pr_2_EuFeO_6_ across the temperature range. The PF of Pr_2_EuCoO_6_ starts increasing beyond 350 K and attains a value of 3.5 × 10^9^ W mk^−2^ s^−1^ at 800 K, while the PF of Pr_2_EuFeO_6_ starts increasing beyond 600 K and reaches 0.8 × 10^9^ W mk^−2^ s^−1^ at 800 K. The elevated power factor of Pr_2_EuCoO_6_ at higher temperatures suggests outstanding thermoelectric performance, which is attributed to the beneficial interplay between the Seebeck coefficient and electrical conductivity. The rise in the PF of both materials as the temperature increases is due to thermally activated carriers. The *ZT* value, also known as the figure of merit, serves as a dimensionless indicator of how effectively a thermoelectric material can convert heat into electricity. Fig. 8(f) displays the computed *ZT* value of both materials. The plots suggest higher values of *ZT* for both materials at room temperature. The *ZT* values of Pr_2_EuCoO_6_ and Pr_2_EuFeO_6_ at room temperature (300 K) are 1 and 0.6, respectively. The *ZT* values of both materials gradually decrease with an increase in temperature and reach 0.7 for Pr_2_EuCoO_6_ and 0.1 for Pr_2_EuFeO_6_ at 800 K. The temperature-dependent *ZT* value of the materials depends on the electrical conductivity (*σ*), the Seebeck coefficient (*S*), and the thermal conductivity (*κ*) of the materials. The different behaviors of Pr_2_EuCoO_6_ and Pr_2_EuFeO_6_ are due to their distinct electronic and thermal properties. The *ZT* suggests that both materials show excellent thermometric performance across the temperature range. These materials have a large number of thermoelectric applications, including temperature sensors,^80^ thermal energy harvesters,^81^ heat capacity-based thermometry,^82^ and thin-film infrared (IR) sensors.^83^

## 4 Conclusion

In summary, this study is carried out using the Wein2K package based on DFT and utilizing mBJ+U approximations to achieve ground state characteristics of Pr_2_EuMO_6_ (M = Co and Fe) perovskites. The structural, magnetic, optoelectronic, and thermoelectric properties of the materials are computed to explore their utilization in different technological applications. The electronic properties suggest that the materials have metallic, up-spin, and semiconducting natures in the down-spin configuration, predicting the half-metallic nature of both materials. Half-metallic materials have a large number of applications, specifically in spintronics. The magnetic nature of the materials is analyzed by performing spin-based computations. The computations predict the magnetic moment of the materials. The computed total magnetic moments of the materials Pr_2_EuCoO_6_ and Pr_2_EuFeO_6_ are 11 (*µ*_B_) and 14 (*µ*_B_), respectively. The integral value of the total magnetic moment indicates the ferromagnetic nature of both materials, which is verified by achieving the most stable volume optimization curve in FM compared to the AFM magnetic phase. The optical characteristics of the materials, including parts of the complex dielectric function, absorption, energy loss, refractive index, and reflectivity, are computed to understand their photonic nature for many potential applications. The study of optical parameters suggests that materials are good choices for photovoltaic and solar cell applications. Thermometric parameters of the materials are calculated (in spin-down) using the BoltzTraP code. Pr_2_EuCoO_6_ has higher electrical and electronic thermal conductivities compared to those of the Pr_2_EuFeO_6_ material at higher temperatures. The Seebeck coefficient of the materials suggests that Pr_2_EuCoO_6_ has an n-type semiconducting nature and Pr_2_EuFeO_6_ has a p-type semiconducting nature. The power factor (PF) of both materials is lower at room temperature and increases linearly with an increase in temperature. The PF of Pr_2_EuCoO_6_ is comparatively higher than that of Pr_2_EuFeO_6_ at higher temperatures. The *ZT* Pr_2_EuCoO_6_ is higher, with a value of 1.0 at room temperature (300 K) and decreases to 0.7 at 800 K, while Pr_2_EuFeO_6_ has a *ZT* value of 0.6 at room temperature and decreases to 0.1 at 800 K. The thermometric study suggests that Pr_2_EuCoO_6_ has a higher thermoelectric performance compared to Pr_2_EuFeO_6_ across the temperature range. The results highlight the future potential of Pr_2_EuCoO_6_ and Pr_2_EuFeO_6_ as viable options for spintronic and thermoelectric applications, attributed to their semiconducting characteristics and moderate thermoelectric performance.

## Author contributions

Ahmad Ali: supervision, conceptualization, investigations, formal analysis, data curation, writing – original draft, and writing – review and editing. Gulzar Khan: software, investigation, data curation, formal analysis, writing – review & editing, and writing – original draft. Tania Gul: formal analysis, data curation, conceptualization, visualization, and writing – original draft. Fareha: investigations, formal analysis, data curation, conceptualization, validation, and writing – original draft. Sikander Azam: methodology, conceptualization, investigations, formal analysis, data curation, writing – original draft, and writing – review and editing. Osama Oqilat: conceptualization, investigations, methodology, writing – review and editing, and writing – original draft. Hijaz Ahmad: conceptualization, formal analysis, data curation, writing – review & editing, and writing – original draft.

## Conflicts of interest

The authors declare that there are no conflicts of interest regarding the publication of this paper. All authors have contributed to this work according to the academic and research standards, and there are no competing interests, financial or otherwise, that could have influenced the outcomes of this study.

## Data Availability

The datasets generated and/or analyzed during this study are available from the corresponding author upon reasonable request.
